# Remote assessment in sport and exercise medicine (SEM): a narrative review and teleSEM solutions for and beyond the COVID-19 pandemic

**DOI:** 10.1136/bjsports-2020-102650

**Published:** 2020-06-30

**Authors:** H Paul Dijkstra, Emin Ergen, Louis Holtzhausen, Ian Beasley, Juan Manuel Alonso, Liesel Geertsema, Celeste Geertsema, Sofie Nelis, Aston Seng Huey Ngai, Ivan Stankovic, Stephen Targett, Thor Einar Andersen

**Affiliations:** 1 Department of Medical Education, Aspetar Qatar Orthopaedic and Sports Medicine Hospital, Doha, Ad Dawhah, Qatar; 2 Department for Continuing Education, University of Oxford, Oxford, UK; 3 Sports Medicine Department, Aspetar Qatar Orthopaedic and Sports Medicine Hospital, Doha, Ad Dawhah, Qatar; 4 Section Sports Medicine, University of Pretoria Faculty of Health Sciences, Pretoria, South Africa; 5 The Royal Ballet, London, UK; 6 Department of Sports Medicine, Oslo Sports Trauma Research Center, Norwegian School of Sport Sciences, Oslo, Norway

**Keywords:** sports and exercise medicine, injury, illness, sports physician, evidence based review

## Abstract

**Background:**

The COVID-19 pandemic forces sport and exercise medicine (SEM) physicians to think differently about the clinical care of patients. Many rapidly implement eHealth and telemedicine solutions specific to SEM without guidance on how best to provide these services.

**Aim:**

The aim of this paper is to present some guiding principles on how to plan for and perform an SEM consultation remotely (teleSEM) based on a narrative review of the literature. A secondary aim is to develop a generic teleSEM injury template.

**Results:**

eHealth and telemedicine are essential solutions to effective remote patient care, also in SEM. This paper provides guidance for wise planning and delivery of teleSEM. It is crucial for SEM physicians, technology providers and organisations to codesign teleSEM services, ideally involving athletes, coaches and other clinicians involved in the clinical care of athletes, and to gradually implement these services with appropriate support and education.

**Conclusion:**

teleSEM provides solutions for remote athlete clinical care during and after the COVID-19 pandemic. We define two new terms—eSEM and teleSEM and discuss guiding principles on how to plan for and perform SEM consultations remotely (teleSEM). We provide an example of a generic teleSEM injury assessment guide.

## Introduction

The rapid spread of the COVID-19 pandemic is forcing clinicians to think differently about providing and continuing care to new and existing patients. Many individual clinicians and hospitals implemented telehealth—in some countries (eg, South Africa) only allowed where an established clinician–patient relationship already exists, with some exceptions (psychology or psychiatry services). Although safe, effective and convenient, there are complex challenges to implementing video outpatient consultations in organisations that are hesitant to change.^1^ A recent paper provided detailed guiding principles for remote assessment in primary care for patients with possible or established COVID-19.^2^ The author emphasised that telehealth is a service and not a technology. The Royal Australian College of General Practice published an online guide supported by flow charts of practical steps to providing telephone and video consultations in general practice.^3^

The clinician’s duty of care when discussing specialties is different from that of primary care. The primary care physician usually has a known patient pool, is the first point of contact and has specific duties, such as screening programmes. Some of this is also true for sports medicine (for instance a team physician), but when practising as a specialist and seeing referred patients only, these differences may have ethical implications in the eHealth context. There are examples of eHealth in musculoskeletal physiotherapy and medical specialties, such as ophthalmology, dermatology, radiology, urology, physical medicine and rehabilitation, and orthopaedics.^4–14^

Sport and exercise medicine (SEM) physicians can learn from these but should also develop and implement their own guiding principles.

## Aim

This paper aims to present some guiding principles on how to plan for and perform an SEM consultation remotely (teleSEM) based on a narrative review of the literature. A secondary aim is to develop a generic teleSEM injury assessment guide.

## Methods

We performed a PubMed and Cochrane Library search on 1 June 2020 using a combination of keywords “telemedicine”, “video”, “sports medicine” and “primary care” for PubMed, and “telemedicine” as keyword for the Cochrane Library, and searched for all types of studies written in English and published since 1 January 2015.

## What is eHealth: the concept and terminology

*eHealth* is deﬁned by the WHO as ‘the practice of medicine and public health supported by electronic processes and communication’.^15^ The Qatar Ministry of Public Health (MOPH) has expanded the WHO definition of eHealth to ‘Transformative and continuous improvement of healthcare through the use of information and technologies that support the delivery of healthcare and clinical research.’^16^ In summary, it brings together people, processes and health services in a collaborative union with a common goal of improving patient care. ‘mHealth’ refers to the application of eHealth using mobile devices.^17^

We define e-Sport-and-Exercise-Medicine (*eSEM*) as the practice of SEM in athlete and public health contexts supported by electronic processes and communication.

The terms telemedicine and telehealth are commonly used and often applied interchangeably. There is, however, a distinction between the two. In short, all telemedicine is telehealth, but not all telehealth is telemedicine.^18–20^

*Telehealth* is a subset of eHealth that includes the delivery of health information, for health professionals and health consumers, education and training of health workers and health systems management through the internet and telecommunications.^18–20^ Ideally, telehealth is the clinical contact component of a comprehensive, population wide eHealth system, such as the Qatar National eHealth & Data Program (QNeDP) of the MOPH.^15^

*Telemedicine,* however, is a subset of telehealth that refers only to the provision of healthcare services and education over a distance, using telecommunications technology.^21^ Telemedicine involves the use of electronic communications and software to provide clinical services such as diagnosis and patient care without an in-person visit.

We define tele-Sport-and-Exercise-Medicine (*teleSEM*) as the use of electronic communications and software to provide clinical SEM services such as diagnosis and patient care without an in-person visit. Like telemedicine, teleSEM is an SEM patient service frequently used for follow-up consultations, management of chronic conditions, medication management, specialist consultation and many other clinical services provided remotely via a secure video, audio connections and mobile phone applications.^18–20^

The teleSEM consultation has three important stages each with its own considerations: (1) planning the consultation, (2) performing the consultation and (3) actions after the consultation (table 1).

**Table 1:** The tele-Sport-and-Exercise-Medicine (teleSEM) process

Planning the remote consultation	Performing the remote consultation	After the remote consultation
Establish the need for a remote consultation Decide on the consultation participants Choose wisely between text, audio or video Know the technology Ensure remote access to the electronic health record Apply ethical guidelines	Have the condition-specific teleSEM guide ready (if you know the type of condition) Connect, introduce yourself (and other team members) and confirm the patient's identity Perform an initial rapid health status assessment Take a history (condition, general, sport, performance goal) Perform a remote SEM physical examination Consider options; discuss a care plan Decision and actions	Accurate and comprehensive notes in the patient's health record Arrange further investigations, follow-up, referral to other members of the multidisciplinary team (physiotherapist, podiatrist, etc), discharge, urgent hospital admission for further care

SEM, sport and exercise medicine.

## Implementing a remote consultation service in SEM

Many SEM physicians and sports medicine organisations are rapidly implementing or considering to introduce eSEM and teleSEM services. It is important to ensure approval and licensing by the involved healthcare organisation, as well as security of personal and medical information exchanged on such platforms. SEM physicians, technology suppliers and organisations should codesign these services, ideally involving athletes, coaches and other clinicians involved in clinical services to athletes. The focus of this paper is on the clinical and interactional aspects of a teleSEM service. The organisational and operational aspects that need to be in place to support an effective teleSEM service are vital and complex, but beyond the scope of this paper.

## Planning a remote consultation

### Establish the need for a remote consultation

The obvious need for a remote clinical consultation exists where a patient needs clinical care but cannot access such a service in person. This might be due to geographical challenges (remote Scotland), safety issues for patient and clinician (war, pandemics), mobility issues (relevant to patient and clinician), or in sports medicine where individual athletes or teams travel to training camps and competitions without medical support staff. It is important to consider what can and what cannot be done in the eHealth context.

### Decide on the consultation participants

Clinicians should exercise care when selecting patients for teleSEM consultations. Shaw *et al* pointed out that such consultations work better when the clinician and patient know and trust each other.^22^ Furthermore, Donaghy *et al* stressed that while there are distinct advantages to this type of consultation, patients that require examination or have complex or sensitive problems (eg, disability) should consult in the traditional face-to-face method.^23^ It may be that the consultation is an initial assessment, but knowledge of the patient’s age,^24^ ethnicity^25^ and gender^26^ are important factors that should, and invariably are, taken into account in any consultation. TeleSEM providers should be sensitive to a patient’s gender preference of the consulting physician. Research in Saudi Arabia by Alyahya *et al* 27 demonstrated that this may be the case. Therefore, it may be prudent to ask the patient at the time of making an appointment, whether they do have a preference. Some consultations may require an interpreter or advocate, provided by someone in the room, or remotely, accessed by dial-in, and with suitable introduction to the patient.^3^ A medical interpreter, a relative or other person who is with the patient can fulfil this role. According to the virtual online consultations: advantages and limitations (VOCAL) study (video consultations in this research), this type of consultation was rated as popular for both patients and staff, although not all staff chose to use it.^22^

The use of multidisciplinary teams has long been lauded in healthcare, and has countless benefits for all concerned in patient care.^28–30^ While not all multidisciplinary team members may be available at any one time, many SEM clinical settings will involve a physiotherapist and a sports physician in consultations.

In addition to an interpreter or patient advocate, a physiotherapist might also be present during a teleSEM consultation. The physiotherapist can advise on appropriate interventions before handing back to the clinician for a recap and the conclusion. Any literature or web/YouTube references can be emailed to the patient at the end of the consultation, with the relevant references detailed in the patients’ electronic health record. An example of the type of interaction is outlined in ‘Improving physiotherapy access using telehealth’.^31^

The SEM physician should keep a checklist of all the members of the consultation team and ensure to introduce all individuals present during the virtual consultation room to each other. Audit and review of these processes, which must include all participants’ views, are integral to the ongoing usability and success of such a programme, as demonstrated in the VOCAL study.^22^

### Choosing wisely between text, audio or video

Evidence for the indications, selection of eHealth type, patient satisfaction and success of clinical outcomes is poor.^32–34^ Patient preference and ability to access technology, patient expectations from the interaction and intended clinical outcomes are some considerations in choosing the medium of choice. Telephone conversations are easy, readily accessible and most people are familiar with them. Telephone conversations are appropriate for emergency (911/999) calls, follow-up of clinical progress and results review for existing patients, and administrative issues such as sick leave arrangements. Video may be less accessible in less-resourced areas, or because of adversity or ignorance of technology. However, some benefits of video consultations are that it adds a visual dimension to the consultation permitting a certain level of enhanced interaction to gauge non-verbal cues and visualise external physical signs.

From the clinician’s perspective, the intended outcome of the teleSEM consultation will direct the decision. The patient should have a choice in the matter and be given an option to select preferences of telephone or video consultation when making online appointments. The selection can also extend to a preferred clinician and type of problem (acute or chronic, body part, etc).

SEM-specific mHealth interactions with athletes and support staff have been in use for years. mHealth (mobile phone, or the practice of medicine via mobile devices) is particularly suited for this purpose. Text messaging with WhatsApp is a cheap and efficient tool for communications in athlete care, for example, between therapists and athletes. Athletes’ rehabilitation progress can be tracked and adapted at any time. SEM physicians can stay up to date and provide feedback on present or new injuries while athletes are away. Health information can be shared in a team environment.^17^ Text messaging is also widely used for administrative athlete medical tasks, such as providing athlete whereabouts to doping control authorities.

### Know the technology

The use of eHealth, specifically telemedicine, is dependent on access to technology in the population where it is applied. More than 52% of the world’s population use the internet, 97​% live within reach of a mobile cellular signal and 93% within reach of a 3G (or higher) network.^35^

Many digital aids are available to assist healthcare provider–patient communication in the telemedicine context. Some of these are integral to larger eHealth systems, for example, the Personal Health Account of the QNeDP. There are purpose-specific apps as seen in the National Health System App Library in the United Kingdom,^36^ dedicated sports medicine apps such as Physiotools and non-specific social media platforms such as VSee, WhatsApp, Skype or Zoom.^37–41^

### Ensure remote access to the (electronic) health record

Some eHealth solutions are integrated with electronic health records. However, not all solutions have this ability and health organisations (whether private practices or large hospitals) must approve of and pay for such integration. Regardless of the status of integration between these two electronic platforms, clinicians must have access to the electronic health record as a prerequisite to providing any eHealth service. The clinician should have independent access to the electronic health record on a different platform if there is no integration through the eHealth solution. Existing health information regarding patients should be at the clinician’s fingertips and any interaction with patients via eHealth services should be documented with the same rigour as a face-to-face consultation. Where paper-based clinical records are used, these must be at hand.

### Apply ethical guidelines

eHealth services provide a continuum of technologies that offer new ways to deliver care. Although fundamental ethical responsibilities apply in every kind of care, the continuum of possible patient–physician/clinician eHealth interactions results in different levels of healthcare provider accountability. The same apply to eSEM and teleSEM.

Healthcare practitioners using eHealth should be guided by core ethical principles (box 1).^42–44^

Box 1 **Core ethical principles for healthcare practitioners providing eHealth services, including e-Sport-and-Exercise-Medicine and tele-Sport-and-Exercise-Medicine.**

**Core ethical principles**

- Ensure patient safety
- Use secure and effective communication methods
- Recommend appropriate and practical treatment options
- Ensure that patient feedback mechanisms are in place
- Implement strategies to evaluate and ensure patient satisfaction

Medical ethics play an important role in adhering to these principles. The five key components of an eHealth medical ethics code include mutual respect, promoting open communication and consent, informed care and shared treatment decisions, access to health information and physician autonomy and responsibilities (table 2).^45 46^

**Table 2:** The five key components of an eHealth medical ethics code

Key components of eHealth medical ethics code	
Mutual respect	The patient–physician relationship must be based on mutual trust, respect and safety. It is therefore essential that the physician and patient be able to identify each other reliably when using eHealth services.^56^
Promoting open communication and consent	An eHealth consultation must be treated like any other outpatient consultation, safeguarding sensitive or confidential information at all times.^57^
Informed care and shared treatment decisions	eHealth consultations are ideal in situations where a physician cannot be physically present in a safe and timely manner. eHealth consultations do not allow for the performance of a physical examination; most non-verbal clues usually present in face-to-face meetings will be absent. These might affect the quality of eHealth communication. The principles of shared decision-making are similar in physical or eHealth consultations. However, it might be more challenging in the eHealth setting to confirm the patient’s understanding of the pathology and treatment options. If there is any doubt, a face-to-face consultation should be offered as an alternative. Inform the patient about the nature and limitations of the eHealth consultation and document informed consent. It remains a vital healthcare provider’s responsibility to consider language barriers and to ensure the right to an interpreter or health advocate.
Access to health information	Patients have the right to access all electronic health record information, unless the attending physician specifically restricts access in consultation with a family representative, legal or surrogate guardian. This can be for medical or legal reasons.
Physician autonomy and responsibilities	The normal ethical and professional standards apply to all aspects of a physician’s practice. A physician should not participate in eHealth services if it violates the country’s legal or ethical framework. Physicians should only practice eHealth in countries/jurisdictions where they are licensed to practice. This is an essential consideration for team physicians when travelling with a team to competitions and training camps to another country. Physicians should also ensure medical indemnity that covers eHealth.

### Generic and condition-specific quick guides for remote consultations

The COVID-19 challenges are unprecedented, and SEM physicians must be agile and flexible to adapt their traditional clinical assessments. It is reasonable to assume that most patients who present for a telemedicine consultation will at some point require a face-to-face clinical consultation. However, depending on the situation or the condition, this face-to-face meeting does not necessarily have to be the initial consultation. Triage in SEM can in most cases be through teleSEM, based on history alone. In case of uncertainty (and where appropriate), initial special investigations may be arranged, followed by a face-to-face consultation for examination and review of the results. It may even be possible to provide initial advice and a rehabilitation plan, followed later by a face-to-face follow-up consultation. While this is a reversal of the usual steps in clinical assessment, it may be the only reasonable way to provide a service in a ‘lockdown’ or similar situation. Patients will generally understand and appreciate this. Many conditions (such as tendinopathies) are more suitable to this type of consultation, where the physical assessment or imaging add very little additional value to the clinical history.

Similarly, teleSEM is ideal to discuss results following the initial face-to-face clinical consultation in cases where the follow-up would not involve further or a repeated patient examination (eg, where there are no contradictory imaging findings).

Team physicians and physicians who work with a specific cohort of athletes can offer teleSEM for routine athlete health monitoring (in those without any new injury or illness), and other situations where the consultation is mostly a history and results review.

A potential new area of SEM eHealth service is a teleSEM consultation for general health advice, not specific to a particular patient or pathology. In such a consultation, an athlete will engage with an SEM physician and ask one-on-one questions about health issues, without requiring a specific clinical examination.

**Figure 1:**
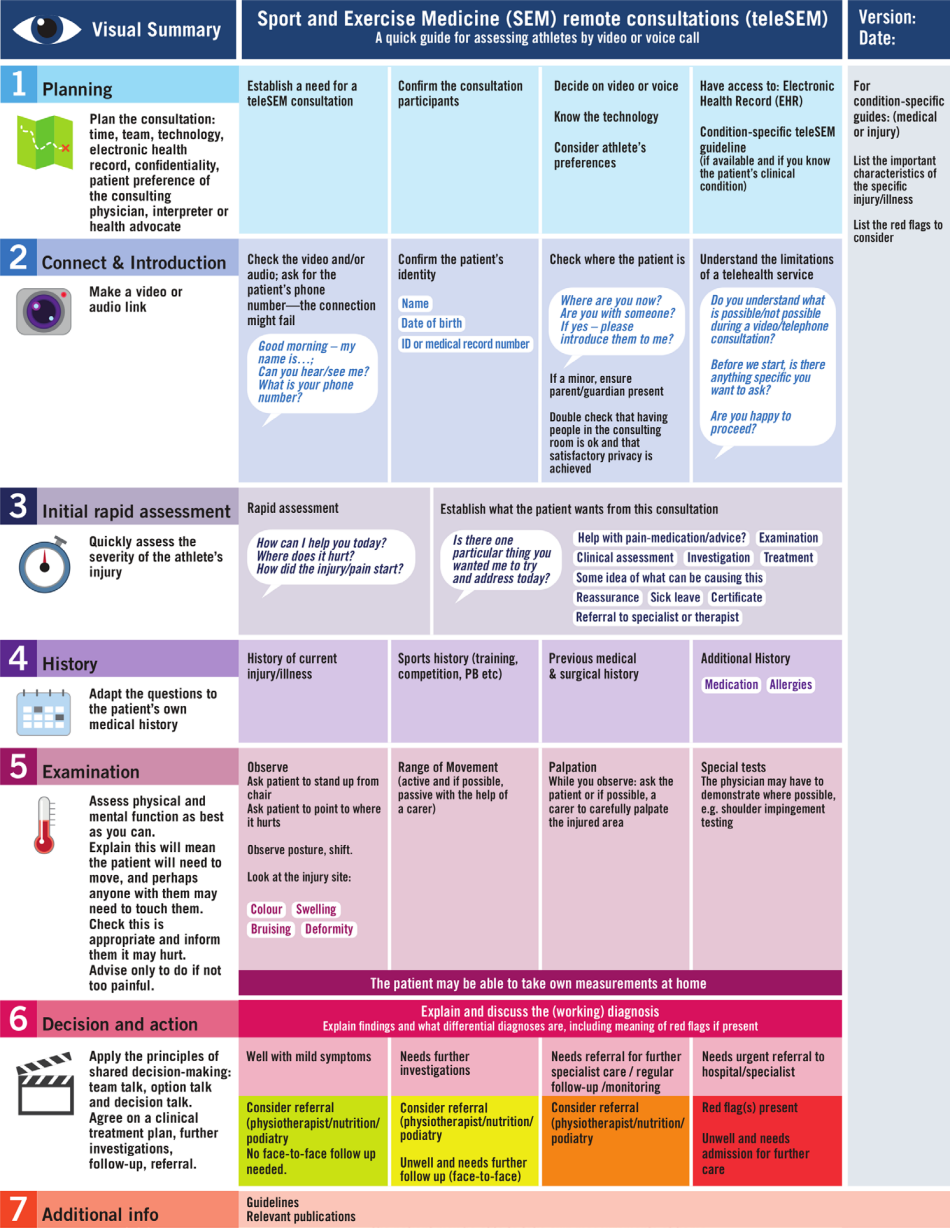
A quick generic sport and exercise medicine (SEM) guide to assessing an athlete with a sports injury remotely (teleSEM; adapted from Ref. 2).

The teleSEM consultation can be facilitated by condition-specific guidelines based on the generic teleSEM template (figure 1). TeleSEM is particularly suited for preventative exercise medicine, including exercise advice for health.

### eHealth considerations for other sports medicine disciplines and athlete health support services

In addition to specialist SEM physician services, the broader field of sports medicine or athlete healthcare services include several other medical specialties (eg, orthopaedics, cardiology, paediatrics, radiology), team physicians, physiotherapy, sport science services (eg, nutrition and psychology), pharmacy, nursing, dentistry and performance coaching for athletes recovering from injury. These specialties should each develop their eHealth guidelines based on what can and cannot be done in the eHealth context for their specialist field. However, an SEM physician-led team approach to developing eHealth services involving all the different specialties involved in sports medicine will facilitate smooth referral pathways and further enhance the patient’s experience. A potentially powerful teleSEM tool is the ‘multidisciplinary consultation’ where a group of practitioners offer a service to the athlete (possibly by using technology that has been designed for online team meetings).

### Fee for service considerations

eHealth billing varies widely depending on the involved government’s regulations and policies.^47 48^ Not every government or private care provider provides reimbursement for eHealth services.^49^ Services might be free, fully covered by the insurer or the patient, with or without a copayment. It is also possible for certain billing codes not to be covered.^50 51^ These factors contribute to the challenges associated with eHealth services.

## The remote consultation

The consulting team should be ready to connect with the patient using the agreed eHealth technology at the time of the scheduled appointment, and with full access to the electronic health record and teleSEM condition-specific quick guides (figure 1).

### Connection and introduction

Introduce yourself, check the video and/or audio connection and ask for the patient’s phone number in case the connection fails. Confirm the patient’s identity (name, date of birth, identity (ID) or medical record number). It is important to confirm that the patient is in a comfortable and quiet place and whether they are alone or with someone they trust. It is also important to briefly check if the patient understands the limitations of teleSEM consultation and that they are happy to proceed.

### Initial rapid assessment

Do a quick assessment of the patient’s general health status: are they injured/ill or less injured/ill; can they walk? Ask the patient what they want from the consultation. This might include a clinical assessment, reassurance or referral, a certificate or health advice.

### History

The teleSEM history-taking structure is similar to a normal face-to-face consultation; a brief history of the current injury/illness, additional history on medication, allergies, previous medical and surgical history, sport performance and training history (recent and past) as well as the patient’s performance goal(s).

### The remote sports medicine physical examination

Assess the patient’s physical and mental function as best as you can. Ask the patient: “Where does it hurt? Can you point/show me?”. Observe for colour, swelling, bruising and any obvious deformity. Test the involved and contralateral joint range of movement (active and if possible, passive with the help of a carer/health advocate). Ask the patient or if possible, a third party to carefully palpate the injured area while you observe. Consider special tests based on the condition and what is possible. The patient may be able to perform their own special test(s) (eg, empty can test for shoulder pain or a flexion adduction internal rotation test for hip pain), or measurements at home (eg, glucose, blood pressure, pulse, step count for the day/past week).

### Agreeing on a care plan including shared decision-making

Shared decision-making is not so much a step as it is a way of conducting a consultation. But it is most tangible in the final step of the consultation, agreeing on a care plan. As in a face-to-face consultation, during the teleSEM consultation, the following steps are important^52^:

- Team talk—inform the patient that a choice must be made, that they may consult with significant others and you are there to support them.
- Option talk—discuss the options and communicate the risks and benefits of each.
- Decision talk—listen to the patient to help them go from preferences to informed decisions.

### Decision and action: working diagnosis and red flags

The working diagnosis and the presence/absence of any red flag symptoms or signs will all determine the treatment plan. This might be further investigations, a follow-up, a referral, discharge with advice or urgent hospital admission for further care.

## After the consultation

Document the teleSEM consultation, including all patient instructions and the agreed care plan, in the electronic health record. Complete the relevant (paper)work for a prescription, a referral for further special investigations, or to another healthcare provider (physician, physiotherapist, podiatrist). Ensure the appropriate arrangements are in place for a follow-up consultation if required.

## Education and future research

It is paramount to support and educate all the stakeholders before and in the early stages of implementation—not only on the technology, but also on the clinical SEM service itself.^53 54^ Excellent evidence-based resources are freely available to support providers, patients and professionals involved in developing and using video consultations.^55^ Collaborative research is needed on effective implementation of teleSEM services, its effect on patient care (clinical outcomes, quality, safety, satisfaction) and its effect on service providers.

## Conclusion

SEM physicians must think differently about patient care in a time of COVID-19 and in the changed world after COVID-19. eHealth, eSEM and teleSEM are important parts of this new world. We define eSEM and teleSEM and discuss the guiding principles on how to plan for and perform an SEM consultation remotely, and how to arrange further care, including follow-up consultations. We discuss the importance of multidisciplinary teleSEM consultations and provide an example of a teleSEM injury assessment guide. This will assist the SEM physician to plan and deliver wise eSEM care to their patients.

**What is already known**

- Telemedicine is a subset of telehealth that refers only to the provision of healthcare services and education over a distance, using telecommunications technology.
- Telemedicine is a clinical service and not a technology.
- Telemedicine is rapidly implemented in practices and hospitals due to the COVID-19 pandemic.
- The organisational and operational aspects that need to be in place to support an effective telemedicine service are vital and complex.

**What are the new findings**

- It is important for sport and exercise medicine physicians to apply some of the clinical and interactional telemedicine lessons from other medical specialties.
- We define tele-Sport-and-Exercise-Medicine (*teleSEM*) as the use of electronic communications and software to provide clinical sport and exercise medicine services such as diagnosis and patient care without an in-person visit.
- Sport and exercise medicine physicians can adapt our generic teleSEM injury assessment guide to their own context.
